# Symbiosis and Dysbiosis of the Human Mycobiome

**DOI:** 10.3389/fmicb.2021.636131

**Published:** 2021-09-22

**Authors:** Kirtishri Mishra, Laura Bukavina, Mahmoud Ghannoum

**Affiliations:** ^1^University Hospitals Cleveland Medical Center, Urology Institute, Cleveland, OH, United States; ^2^Case Western Reserve University School of Medicine and University Hospitals Cleveland Medical Center, Cleveland, OH, United States; ^3^Center for Medical Mycology, and Integrated Microbiome Core, Case Western Reserve University School of Medicine and University Hospitals Cleveland Medical Center, Cleveland, OH, United States; ^4^Department of Dermatology, Case Western Reserve University School of Medicine and University Hospitals Cleveland Medical Center, Cleveland, OH, United States

**Keywords:** dysbiosis, mycobiome, health, disease, microbiome, commensals

## Abstract

The influence of microbiological species has gained increased visibility and traction in the medical domain with major revelations about the role of bacteria on symbiosis and dysbiosis. A large reason for these revelations can be attributed to advances in deep-sequencing technologies. However, the research on the role of fungi has lagged. With the continued utilization of sequencing technologies in conjunction with traditional culture assays, we have the opportunity to shed light on the complex interplay between the bacteriome and the mycobiome as they relate to human health. In this review, we aim to offer a comprehensive overview of the human mycobiome in healthy and diseased states in a systematic way. The authors hope that the reader will utilize this review as a scaffolding to formulate their understanding of the mycobiome and pursue further research.

## Introduction

The role of commensal and pathologic bacteria in influencing human health has been studied for over a century with numerous direct correlations to disease states as well as symbiotic conditions (Ubeda et al., 2017; Ranjan and Dongari-Bagtzoglou, 2018; Libertucci and Young, 2019). While we have made great strides in our understanding of bacteria, our understanding of fungal commensalism remains limited. Not until recently were fungi thought to be a crucial aspect of the human microbiome, now individually referred to as the mycobiome. Furthermore, due to increasing utilization of antimicrobials and the resultant damage they cause to our microbiome, we are just starting to appreciate the beneficial roles of microbes. With increased understanding of the impact that microbes have in the human physiology and pathology, and advancing technology, there has been an emphasis on exploring the interplay between bacterial and fungal communities as it relates to human health (Ghannoum, 2016).

**FIGURE 1:**
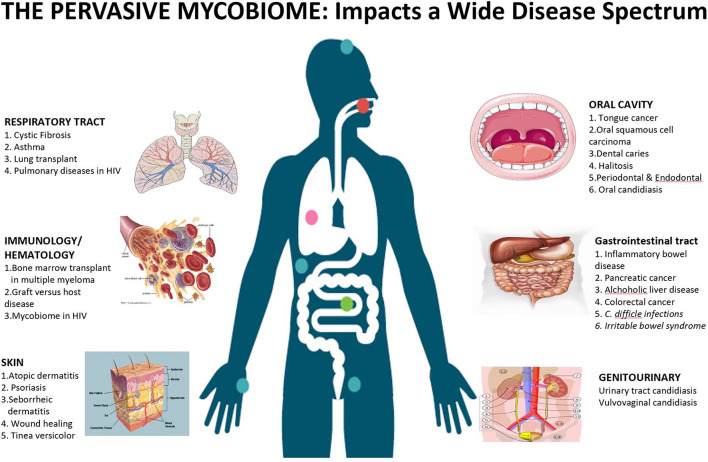
Mycobiome affects a wide spectrum of diseases across various niches in the human body. The cooperative evolution of fungi and bacteria is a complex interplay, where it may be mutualistic in some conditions, while antagonistic in others.

While bacteria comprise the overwhelming majority of biodiversity (>99%) in humans, the shear sizes of the fungal cells, relative to their bacterial counterparts, compensate for this difference (Seed, 2014). Previously, fungi were considered to be irrelevant when they were co-isolated with bacteria, or were considered to be environmental contaminants (Brown et al., 2012; Huffnagle and Noverr, 2013). Now we understand that fungal flora is crucial in maintenance, metabolism, and immunity of the microbiota and the host (Qin et al., 2010). Healthy state is an interplay between an intact immune system, genetic polymorphisms, and microbiota (Iliev et al., 2012). This interplay between bacteria, fungi, and the host are integral in the symbiosis and dysbiosis seen in human health (Figure 1). In this comprehensive review, we will explore the mycobiome present in humans that contribute in a symbiotic manner, as well as the alterations in diversity that may lead to dysbiosis and pathologic states.

## Advances in Technology to Evaluate Mycobiome

Traditionally, fungi have been evaluated using culture-based protocols; however, with advances in deep-sequencing technologies, investigators are able to study fungi that were previously unculturable in a lab. These developments have exponentially accelerated our understanding, and have led to the “human microbiome project,” which focused on the bacterial community, an undertaking that was initiated over a decade ago (Turnbaugh et al., 2007). With increased knowledge, we come to appreciate the complexity of fungi in a more detailed and comprehensive manner. Furthermore, we are able to evaluate the inter-kingdom interactions to improve our understanding of the microbial diversity and the complex network of species that yield the healthy and pathologic states.

**FIGURE 2:**
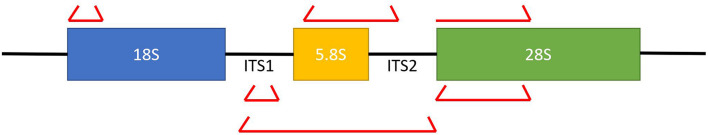
A diagrammatic representation of the various fungal ribosomal genes that can be targeted for sequencing. The ITS sequences provide the best balance of conservation which facilitates targeting, while also providing diversity to differentiate between organisms. The red brackets represent primer sequences that can be utilized to sequence the desired region.

In comparison to the Sanger sequencing method, next generation sequencing allows us to perform community-level analyses in a more efficient and cost-effective manner (Ackerman and Underhill, 2017). Multiple gene loci have been targeted for fungal gene profiling including 18S ribosomal rRNA (small subunit) and 28S rRNA region (large subunit), along with the two internal transcribed spacer units 1 and 2 (ITS1/ITS2) (Dollive et al., 2012). The small subunit is highly conserved and lacks discriminatory power, while the larger subunit is more hypervariable in comparison to the 18s ribosomal rRNA. Overall, the ITS1 and ITS2 are used most commonly to detect fungi as it has the best combination of variability to identify species while also conserving targets that can be readily targeted (Figure 2; Dollive et al., 2012; Findley et al., 2013). The ITS sequences are aligned to the operational taxonomic units (OTUs) in fungal databases to identify species. A standing challenge with further examination of the mycobiome remains the abundant fungal diversity in the environment; however, with concerted efforts we are able to identify approximately 80% of the mycobiome found in fecal and mucosal samples (Findley et al., 2013; Underhill and Iliev, 2014; Tang et al., 2015; Motooka et al., 2017). Despite this, our understanding of the fungal world in limited due to their vast diversity with limited effort from the scientific community, variations in the DNA extraction techniques, and lack of well-maintained and comprehensive database for sequence comparison.

## Significance of Mycobiome in Cross-Kingdom Interactions

As mentioned previously, there is increased appreciation for inter-kingdom interactions between bacteria and fungi as they relate to human health. For example: there is increasing evidence that infections may frequently be co-inhabited by fungi and bacteria. This is certainly true in cases of wound infections, but also in cases of “isolated” fungal infections. Various studies have found reported cases of candidemia to have mixed microbial infections up to 38% of the time (Hermann et al., 1999; Klotz et al., 2007; Kim et al., 2013). In particular, our experiences with chronic wound patients have suggested that bacteria and fungi co-inhabit the wound bed to increase pathogenicity and tolerance of antibiotics (Ghannoum, 2016).

**FIGURE 3:**
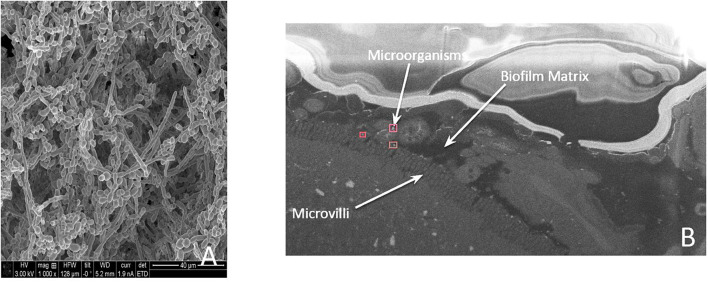
**(A)** A microscopic image of an in vitro biofilm. **(B)** A medial section of a mouse colon demonstrati ng microorganisms present on the villi under a protective barrier formed by the biofilm matrix.

In a 2016 study by Kalan et al. (2016), the authors utilized an *in vitro* model to demonstrate a close interaction between bacterial and fungal cells, with yeast cells forming the biofilm core, and bacteria forming the biofilm periphery. In this model, the fungi benefit by obtaining virulence factors (i.e., the ability to form hyphae and the ability to secrete extracellular enzymes), while the bacteria gain antibacterial tolerance provided by the protective fungal matrix (Hoarau et al., 2016; Kalan et al., 2016). Similar interplay has been reported with *Escherichia coli*/*Staphylococcus aureus* and *Candida albicans* (De Brucker et al., 2015; Kong et al., 2016). Our group has demonstrated a cooperative survival technique utilizing mixed biofilms between the bacteriome and the mycobiome in Crohn’s disease (CD) patients, where we see inter-kingdom association between *Candida tropicalis* and *Serratia marcescens*/*E. coli* (Hoarau et al., 2016). Figure 3 provides an *in* vitro and *in vivo* representation of this finding. These findings have also been demonstrated by Sokol et al. (2017), who show positive correlation between *Saccharomyces* and *Malassezia* with various bacterial species in CD. Similarly, patients with cystic fibrosis (CF) who suffer from *Psuedomonas aeruginosa* infection are frequently found to have persistent infections from *Aspergillus fumigatus* or *C. albicans* compared to patients without these fungi (Amin et al., 2010; Chotirmall et al., 2010). These findings further reinforce our appreciation of the inter-kingdom interplay that may need to be addressed in treating complex chronic wounds, patients with severe CD, or patients with CF who suffer recurrent pulmonary infections.

In contrast to synergistic interactions highlighted above, microbes may also interact in an antagonistic manner, where one organism may inhibit the growth of another organism (Brogden et al., 2005; Rall and Knoll, 2016). To add another layer of complexity, the host and the environment may further impact the proliferation or inhibition of microbial growth. These factors dynamically influence the synergistic or antagonistic influence of various microbes (Wargo and Hogan, 2006; Kapitan et al., 2018). The prevalence of certain organisms and the variations in biodiversity may lead to a state of dysbiosis in the host, which may make the host vulnerable to various pathologic conditions. Kruger et al. (2019) aptly describe the interactions between fungi and bacteria as one of five modes: (1) physical interaction, (2) environmental interaction, (3) biofilm formation, (4) chemical and metabolic interaction, and (5) competitive interaction.

A unique challenge with studying the bacterio- and the myco-biome simultaneously is the difficulty with extracting nucleic acids from these species without a biased protocol. The rigid cell walls of fungi, and the mechanical, chemical, and enzymatic steps needed to effectively retrieve nucleic acids from bacteria can inherently introduce bias into the protocol (Wesolowska-Andersen et al., 2014; Vesty et al., 2017). Consequently, most protocols are targeted at identifying specific species, which may not permit other species to be properly amplified using nucleic acid based assays (Underhill and Iliev, 2014). This challenge leads to variations in the micro- and mycobiome analysis between individuals and protocols. To date there is no established gold standard protocol to evaluate the inter-kingdom interactions between bacteria and fungi (Tang et al., 2015; Enaud et al., 2018). To complicate matters further, there are often transient colonizers in various niches, which further skew genetic analyses or culture results (Chen et al., 2011). All of these factors combined, lead to a consistent challenge and require a disciplined approach to studying the microbial flora in human physiology and disease states. While there remains variability in findings in this domain, there is little debate that the interplay between bacteria and fungi are crucial to a comprehensive understanding of the human health (Brogden et al., 2005; Wargo and Hogan, 2006; Shirtliff et al., 2009; Leclair and Hogan, 2010; Morales and Hogan, 2010; Peleg et al., 2010; Peters et al., 2012; Allison et al., 2016; Forster et al., 2016; Rall and Knoll, 2016; Kapitan et al., 2018).

## Mycobiome in Healthy and Diseased States

With the stated limitations in mind, we will explore various niches within human health and characterize the normal fungal flora. Furthermore, we aim to comment on the fungal and bacterial interactions in these domains during a healthy state, and the alterations occurring in dysbiotic states that may lead to pathologic conditions.

### Gastrointestinal Mycobiome

#### Oral Mycobiome

The oral cavity is the initial contact of the human gastrointestinal flora to the environment (Dewhirst et al., 2010). This cavity is a unique reservoir for microbial growth due to the various surfaces present. For example: the microbes on the tongue, a mucosal surface with constant shedding, is drastically different than the teeth, a surface that represents a prime estate for biofilm formation (Arweiler and Netuschil, 2016). As mentioned previously, biofilm are three dimensional structures comprised of bacterial and fungal biomass that increases microbial pathogenicity and tolerance to antibiotics (Costerton et al., 1999). This may lead to pathologic states of gingival plaques and cavities (Zhang Y. et al., 2018).

Compared to the bacteriome, the mycobiome of the oral cavity is vastly understudied. The most common fungal organisms identified in the human oral cavity are *Candida* (with *C. albicans* as the most dominant species), *Cladosporium, Aureobasidium, Saccharomycetales, Aspergillus, Fusarium, Cryptococcus*, and *Malassezia* (Ghannoum et al., 2010; Dupuy et al., 2014). Once again, the protocols utilized to characterize the biodiversity in the oral cavity leads to inherent variations in the species. In fact, some studies suggest that not only is there significant microbial variability between individuals, but there can be intrapersonal variability in biodiversity through the course of a day (Costello et al., 2009; Caporaso et al., 2011; Stahringer et al., 2012; David et al., 2014a; Ding and Schloss, 2014; Flores et al., 2014; Cameron et al., 2015; Belstrom et al., 2016; Utter et al., 2016). This was further demonstrated within the mycobiome between individuals; however, the intrapersonal fungal diversity remained relatively stable over the course of a 30 day period (Monteiro-da-Silva et al., 2014).

In states of dysbiosis, the fungal and the bacterial biodiversity may be compromised (Selwitz et al., 2007; Takahashi and Nyvad, 2011; Zhang Y. et al., 2018). Once again, the interplay between the bacteriome and mycobiome is critical in these scenarios. For example: a frequent intake of carbohydrates combined with poor oral care may lead to acidification of the niche due to sugar-fermentation and acidification from the bacteria. This change in pH may provide a selective pressure in which certain microbes may thrive while others may perish (Takahashi and Nyvad, 2011). Similarly, exposures to antibiotics or states of immunodeficiency may provide a selective pressure that leads to a decrease in biodiversity while allowing organisms such as *Candida* to overgrow and proliferate (Costa et al., 2013; Chanda et al., 2017). This state may lead to individuals suffering from oral candidiasis or thrush (Millsop and Fazel, 2016). Interestingly, many newborns who have not achieved adequate biodiversity suffer from thrush, which further implicates the need for microbes in maintaining a competent immune system to keep these organisms under control (Dickstein, 1964; Hoepelman and Dupont, 1996; Jain et al., 2010; Sampaio-Maia and Monteiro-Silva, 2014; Dzidic et al., 2018).

Gingivitis is another condition associated with oral dysbiosis, which may further progress to periodontitis after a period of chronic inflammation (Zhang Y. et al., 2018). Various fungal organisms have been identified in patients suffering from this condition (Hajishengallis, 2014). Specifically, *Candida* and *Rhodoturula*, were found in many such patients; however, only *C. albicans* was found in all patients (Canabarro et al., 2013). Furthermore, *C. albicans* was most frequently found in patients with bacterial species that operate in mutualistic relationship to increase pathogenicity and lead to virulent plaque formation and increase disease severity (Gross et al., 2012). The two most common bacterial species identified were *Streptococcus mutans* and *Streptococcus viridans*. Focusing on *S. mutans*, this species of bacteria is commonly found in biofilms formed on teeth (Gross et al., 2012; Metwalli et al., 2013). The pathogenic potential of this organism is believed to arise from the formation of extracellular polysaccharides (EPS) by utilizing an exoenzyme called glucosyltransferase (Falsetta et al., 2012, 2014; Metwalli et al., 2013). This enzyme forms biofilms by adhering to surfaces, and ultimately creating an acidic environment, which leads to the formation of dental plaques (Forssten et al., 2010; Metwalli et al., 2013). An *in vitro* study demonstrated that presence of *C. albicans* leads to increased expression of glucosyltransferase B, which in turn binds to the surface of *C. albicans* and leads to formation of extracellular matrix (Falsetta et al., 2014). Furthermore, EPS also sequesters antifungal drugs such as fluconazole, which renders the *C. albicans* tolerant to the medication (Kim et al., 2018). Lastly, fungal products such as farnesol lead to *S. mutans* proliferation in high concentration (Kim et al., 2017).

This mutualistic relationship is challenged by Willems et al. (2016), who argue that *C. albicans* actually increases the pH, which would inhibit *S. mutans* proliferation. This has been further supported by Barbosa et al. (2016), who found reduced hyphal formation in *C. albicans* in larval tissue when co-injected with *S. mutans*.

Despite the conflicting conclusions of the interplay between *C. albicans* and *Streptococcus mutans*, there are various other organisms that are known to operate synergistically with *C. albicans* in infections, and oral candidiasis (Diaz et al., 2012). *Streptococcus oralis*, *Streptococcus sanguinis*, and *Streptococcus gordonii* have been associated with potentiating *C. albicans* infections and leading to a deep organ dissemination of this pathogenic yeast (Diaz et al., 2012; Xu et al., 2016). In addition, *C. albicans* has been found to potentiate the growth of anaerobic bacteria in aerobic conditions to exacerbate gingivitis (Karkowska-Kuleta et al., 2018). Furthermore a simultaneous *C. albicans* infection in an individual with oral lichen planus can lead to exacerbation of symptoms in these individuals who suffer from a chronic inflammatory condition. The diagnosis and treatment can be challenging in these patients, as the typical treatment for lichen planus is steroids, but it may promote further confluence of the *C. albicans* (Karkowska-Kuleta et al., 2018).

Overall, the impact of oral mycobiome in oral pathologic conditions is incompletely understood, but there is mounting evidence that fungal organisms play a critical role in potentiating disease (Janus et al., 2017). In addition, most studies are performed with limited number of organisms, which is another shortcoming of these studies, as the full complexity of a biodiverse environment cannot be entirely evaluated (Janus et al., 2017).

#### Intestines

The microbiome of the gut is well characterized compared to others organs due to the ability to readily evaluate this flora via fecal samples (Durban et al., 2011; Carstens et al., 2018; Altomare et al., 2019). However, this methodology is more indicative of the intraluminal organisms rather than the mucosal or the tissue specific microbiota (Zoetendal et al., 2008; Nash et al., 2017). Despite the stated limitation, the mucosal microbiome has been studied extensively in patients with inflammatory bowel disease (IBD) in whom tissue from biopsies and surgeries are routinely collected (Lepage et al., 2005; Sepehri et al., 2007; Ott et al., 2008; Altomare et al., 2019). Similar to the oral microbiome, there is interpersonal variations in the gut flora, which can be influenced by various factors such as diet, gender, age, concurrent comorbidities, medications, and environment (Wu et al., 2011; David et al., 2014b; Theriot et al., 2014; Strati et al., 2016). In addition, characteristics inherent to the intestinal environment such as pH, oxygen level, and the presence of macro- and micronutrients may impact the biodiversity and floral density reffered to as abundance (Qin et al., 2010; Donaldson et al., 2016).

It is estimated that 0.1% of the total gut microbiome is comprised of fungi (Qin et al., 2010; Ianiro et al., 2016). Some of the major fungal taxa identified in healthy individuals include *Ascomycota* and *Basidiomycota* (Ott et al., 2008; Hoffmann et al., 2013; Nash et al., 2017). Other genera may include *Saccharomyces*, *Candida*, *Malassezia*, and *Cladosporium* (Ott et al., 2008; Hoffmann et al., 2013; Nash et al., 2017). Once again, the interplay between bacteria and fungi are integral to maintaining a healthy state. Multiple studies have demonstrated dysbiosis in pathologic states, especially IBD (Scupham et al., 2006; Dollive et al., 2013; Hoarau et al., 2016; Wheeler et al., 2016; Sokol et al., 2017; Angebault et al., 2018; Kalyana Chakravarthy et al., 2018; Skalski et al., 2018; Sovran et al., 2018; El Jurdi et al., 2019). In a study of patients with CD, there was a close association between pathogenic bacteria and the fungus *C. tropicalis*, with a lower prevalence of beneficial bacteria (Hoarau et al., 2016). Sokol et al. (2017) demonstrated an imbalance of *Basidiomycota/Ascomycota* ratio in patients with IBD. In fact, dysbiotic states in the intestines have been correlated to the onset of CD(Ott et al., 2008; Hoarau et al., 2016; Sokol et al., 2017).

The study of the intestinal microbiome serves as a great opportunity to discuss the concept of co-occurrence/co-exclusion. Patterns of co-occurrence have traditionally been explained by species that prefer similar environmental attributes and exhibit similar tolerances such as pH, temperature, habitat, and biogeographic distribution. This may include interactions that may be mutualistic or parasitic. Identifying co-occurrence may shed light on essential interactions that impact the ecosystem and the biodiversity. These patterns have been studied both at the macro- and the microbiome level. The human genome project has shed light on the interactions within the human gut and oral flora (Ott et al., 2008; Durban et al., 2011; Hoarau et al., 2016). As the reader progresses through the rest of this manuscript, they should attempt to contextualize these interactions to improve their understanding of the human mycobiome.

Fungal prevalence in the gut can be affected by administration of antifungal medications. In a study by Wheeler et al. (2016), it was demonstrated that *Malassezia* and *C. albicans* have a regulatory role in preventing infections from opportunistic organisms such as *Aspergillus amstelodami*, *Epicoccoum nigrum*, and *Wallemia sebi*. These opportunistic pathogens may thrive in a state of dysbiosis initiated by the administration of fluconazole, as demonstrated in a murine model (Wheeler et al., 2016). Ultimately, this imbalance led to acute or chronic colitis in the murine model used. This beneficial effect of fungi was further illustrated by Jiang et al. (2017), where they demonstrated that commensal fungi such as *Saccharomyces cerevisiae*, *C. albicans*, or fungal cell wall mannans may recapitulate the protective benefits of intestinal bacteria.

In a study evaluating the beneficial effects of fungi, *S. cerevisiae* (baker’s yeast), a commonly studied fungi, was used as a probiotic to prophylactically prevent *Clostridium difficile* infections (Massot et al., 1984). In another porcine study, *S. cerevisiae* was able to reduce the translocation of enterotoxigenic *E. coli* (*ETEC*) to confer mucosal immunity (Lessard et al., 2009). The competitive inhibition towards *C. difficile* demonstrated by *S. cerevisiae* does not hold true for other fungal species. Studies examining the protective effects of candidal species on preventing *C. difficile* related colitis, and mortality have yielded conflicting conclusions. Zuo et al. found that high abundance of *C. albicans* was correlated with more severe *C. difficile* infection in a murine model (Zuo et al., 2018). Meanwhile, Markey et al. found *C. albicans* to serve a protective role in their mouse model (Markey et al., 2020). They concluded that *C. albicans* co-inhabitation led to an increased diversity in the overall microbiota, which limited the severity of disease cause by *C. difficile*.

Another notable organism present in the gut that interacts with *C. albicans* is *Enterococcus faecalis* (Allison et al., 2016). In previous studies, *E. faecalis* has been cited to have potentiating effect on *C. albicans* and thought to promote hyphal formation in endotracheal biofilms (Adair et al., 1999). However, in the gut, *E. faecalis* is an inhibitory organism to *C. albicans* (Cruz et al., 2013; Graham et al., 2017). These findings emphasize the variability in inter-kingdom interactions among these organisms based on their niche.

Similar to the dysbiotic effects of antifungal usage, indiscriminate use of antibacterial medications may lead to increased fungal abundance and disseminated candidiasis (Kennedy and Volz, 1985; Mason et al., 2012). In a murine study, the administration of antibiotics led to overwhelming colonization by *Candida* species, while depleting the abundance of Bacteroidetes and Synergistetes (Erb Downward et al., 2013). It appears that the presence of Bacteroides and Firmicutes are critical to regulating candidal confluence (Erb Downward et al., 2013). Overall, *Candida* colonization in the gut can be both beneficial or detrimental. It is beneficial in circumstances when it prevents the overcolonization of other organisms and aids in breakdown of food in collaboration with bacteria. Specifically, *Candida* can breakdown complex carbohydrates leading to by-products that can be metabolized by *Prevotella* (Kraneveld et al., 2012). Which in turn produce by-products that can be used by *Candida*. However, it can be detrimental in states where *Candida* can become overwhelmingly abundant and limit biodiversity.

### Lungs

The advancements in culturing techniques and development of next generation sequencing has debunked the previously held idea that the lungs were sterile (Mitchell and Glanville, 2018). Recent studies have demonstrated that much like the gut, the lungs provide a unique habitat for select organisms to thrive. However, compared to the gut, the overall bioburden in the lungs is significantly lower (Budden et al., 2017). Environmental factors that impact organisms in the lungs are pH level, temperature, and oxygen concentration (Ingenito et al., 1987). Similar to other organ systems, there is variability within varying parts of the lungs themselves based on the variations in the factors listed above (West, 1978).

The most common fungi identified in the lungs of healthy subjects are *Aspergillus, Candida, Penicillium, Clavispora*, and Davidiellaceae (Charlson et al., 2012; van Woerden et al., 2013). In addition, many healthy individuals are colonized by *Pneumocystis* which is of low pathogenic potential in immunocompetent patients; however, it may lead to pneumonia in immunocompromised individuals such as HIV infected patients (Peterson and Cushion, 2005; Montes-Cano et al., 2007; Sethi and Murphy, 2008; Chabe et al., 2011; Huang et al., 2011).

The study of pulmonary microbiome is nascent; however, there is increasing evidence that the interplay between fungal and bacterial species is crucial in dysbiosis. Majority of literature in this domain is based on findings in patients with CF (Cui et al., 2014; Marsland and Gollwitzer, 2014; Mitchell and Glanville, 2018). Due to a defective CF transmembrane conductance regulator protein, these patients form copious amounts of thick mucus in their lungs (along with pathologic manifestations in other organs) which promotes microbial growth and colonization. Furthermore, poor penetration into the viscous mucus prevents adequate treatment with antimicrobials (Robinson et al., 1999). Co-colonization by multiple species has been documented and leads to higher morbidity and mortality in patients with CF (Peleg et al., 2010). Specifically, bacteria such as *P. aeruginosa* and *S. aureus*, along with fungi such as *A. fumigatus* and *C. albicans* are commonly isolated in these patients (Delhaes et al., 2012).

Interestingly, the lungs were one of the first niches where the co-inhabitation of fungi and bacteria was investigated (Kerr et al., 1999). The Gram-negative bacteria, *P. aeruginosa* has been extensively studied as a common cause of infection in patients with CF (Gordee and Matthews, 1969; Kerr, 1994; Hogan et al., 2004; Mowat et al., 2010). Furthermore, the interaction between *P. aeruginosa* and *A. fumigatus* and *C. albicans* have been evaluated in both *in vitro* and *in vivo* models (Kerr et al., 1999; Briard et al., 2016; Chen et al., 2018). The findings from these studies further highlight our previous point of the infancy of microbial interactions as they affect human health and disease.

Despite a dedicated effort in evaluating the impact of *P. aeruginosa* on human health, the *in vitro* and *in vivo* models conclude opposite interactions between the listed organisms. The *in vitro* studies concluded that *P. aeruginosa* inhibits the growth of *A. fumigatus* and *C. albicans* by decreasing filamentation, biofilm formation, and conidia biomass (Mowat et al., 2010). This inhibitory effect is also seen with other fungi that are commonly isolated in the lungs of CF patients (Gordee and Matthews, 1969; Hogan et al., 2004; Kaur et al., 2015; Sedlacek et al., 2015). In return, *A. fumigatus* has the ability to produce gliotoxin which prevents bacterial biofilm formation by *P. aeruginosa* along with other organisms (Reece et al., 2018). The exact mechanism of this antagonism is not fully understood (Chen et al., 2018).

In contrast to the *in vitro* findings, studies using *in vivo* murine models show a synergistic relationship between *P. aeruginosa* and *C. albicans* (Kerr et al., 1999; Hogan et al., 2004; Morales and Hogan, 2010; Roux et al., 2013). This is validated clinically in patients with CF, who have significantly worse clinical course when colonized by two organisms rather than one (Leclair and Hogan, 2010). This points to the major limitation in our understanding of the microbiome as the complexity of host, pathogen, and resources cannot be adequately simulated in an *in vitro* model. It is hypothesized that these organisms synergistically facilitate each other’s growth by inhibiting the action of the alveolar macrophages (Ader et al., 2011; Roux et al., 2013). This was further supported by a clinical study demonstrating prolonged hospitalization in patients with polymicrobial pneumonia, as compared to an infection caused by a single organism (Azoulay et al., 2006).

Another well-investigated model evaluating the interaction between bacteria and fungi involve *Klebsiella* species (Kollef et al., 2005). *Klebsiella pneumoniae* are routinely identified in ventilator-associated pneumonia (Kollef et al., 2005). These species are also found in the gut and lungs of healthy individuals. Nogueira et al. (2019) studied the interaction of *Klebsiella* with *Aspergillus* species and found that the bacteria prevent hyphal formation and spore germination in the fungal organism. The more important finding in the study was identifying the importance of physical contact between the two species that leads to the inhibitory effect (Nogueira et al., 2019). *Klebsiella* also prevents biofilm formation by the *Aspergillus* species, which further inhibits fungal growth (Fox et al., 2014). But similar to previous examples, there is contradictory evidence when the organisms in the model are changed. For example: Frases et al. (2006) demonstrated a synergistic relationship between *Cryptococcus neoformans* and *Klebsiella*. Therefore, it is not possible to generalize the interactions between species, and isolated models may not fully capture the complexity of inter-kingdom interactions. Increased understanding in this domain has clinical implications as immunocompromised patients may suffer from pathologic infections from species such as Mucorales, while the ability to decrease fungal virulence by utilizing another competitive species may offer a therapeutic modality (Ibrahim et al., 2008; Kobayashi and Crouch, 2009; Hover et al., 2016).

### Skin

The human skin is colonized by bacteria, fungi, viruses, and parasites. In context of this review, the majority of studies evaluating cutaneous mycobiome have looked at patients with atopic dermatitis, seborrheic dermatitis, dandruff, or dermatophytosis (Gupta et al., 2004; Havlickova et al., 2008; Seebacher et al., 2008; Gaitanis et al., 2012; Clavaud et al., 2013). Overall, most of the fungi found on the skin are commensal organisms; however, some may harbor pathologic potential (Leung et al., 2016). The pathologic manifestations from these organisms mostly occur in patients who are immunocompromised.

While culture based studies have been used in the past to characterize the skin flora, these studies are unable to fully capture the complexity of the microbiome as well as the interactions that occur between the numerous organisms co-colonizing the skin (Seebacher et al., 2008). The predominant organisms identified on the skin of healthy individuals are members of the *Malassezia* genus, with less common species including *Aspergillus*, *Candida*, *Saccharomyces*, and *Epicoccum* (Seebacher et al., 2008; Clavaud et al., 2013). The fungal skin mycobiome differs with age as younger individuals have greater fungal diversity and less *Malassezia* cultured compared to their adult counterparts (Seebacher et al., 2008). The increase in *Malassezia* occurs sharply around adolescence which may be linked to the change in sebaceous activity. Consequently, availability of lipids provides a needed nutrient for favorable growth of this yeast as most species lack genes for fatty acid synthesis (Jo et al., 2017).

The identification of *Malassezia* has provided insight into various dermatologic conditions such as tinea versicolor, atopic dermatitis, seborrheic dermatitis (Gupta et al., 2004; Gaitanis et al., 2012). Seborrheic dermatitis is a great example to illustrate the possible implications of fungi in cutaneous manifestations and subsequent therapeutic intervention.

Seborrheic dermatitis is a common skin condition that is associated with the scalp and with *Malassezia* as the etiologic agent (Shuster, 1984; Pierard et al., 1997; Nakabayashi et al., 2000). Decreased immune status is associated with the disease, suggesting that immunity may play a significant role in predisposing individuals to seborrheic dermatitis. Antifungal shampoos have a profound impact on the condition, implicating fungi as an important player in the progression of the disease. Because of its connection to sebaceous-dependent organisms, it often starts with adolescents (White et al., 2014). In this population, the disease is linked to dandruff and may have severe manifestations (White et al., 2014). Moreover, *Malassezia globosa* and *Malassezia restricta* are often isolated from patients (Pedrosa et al., 2014; White et al., 2014). *M. globosa* and *M. restricta* are both common components of the skin mycobiome, indicating they are not necessarily the initial cause of the infection. Mycobiome analysis showed a decrease in fungal diversity, with an increase in the Basidiomycota phylum (Park et al., 2012). Additionally, there is a significant decrease in *Cryptococcus* and *Didymella*, accompanied by a significant increase in *Filobasidium floriforme, Malassezia*, and *Penicillium* (Park et al., 2012).

As the largest organ and the most readily accessible organ in the human body, the skin will be a centerpiece of mycobiome analysis as we move forward. The advancing technology will certainly offer us greater insight into the complex interaction amongst the various organisms that are present on the skin. Moreover, unpublished data from our team shows that interactions between *Alternaria* and *S. aureus* occurs in the setting of atopic dermatitis (data not shown).

### Genitourinary Tract

#### Vagina

The microbiome of the vagina is influenced by multiple host and environmental factors such as age, menstruation, pregnancy, health status, hygienic practices, and medications. For example: during reproductive years there is a high supply of glycogen and nutrients which leads to a high confluence of anaerobic organisms, which create an acidic niche (Bradford and Ravel, 2017; Fuochi et al., 2017; Vaneechoutte, 2017; Greenbaum et al., 2019). *Candida* is the most commonly identified vaginal fungus, followed by Saccharomycetales, Davidiellaceae, *Cladosporium*, and *Pichia* in young women (Drell et al., 2013).

One of the most well-studied inter-kingdom interactions in the human body is between *Candida* and *Lactobacilli* (Sobel et al., 1998; De Bernardis et al., 2018). Vulvovaginal candidiasis is a common manifestation of dysbiosis that affects 75% of women at some point through their lifetime (Sobel et al., 1998). Most frequently this condition is caused by *C. albicans*, but may also result from *Candida glabrata*, *Candida tropicalis*, and *Candida parapsilosis* (Sobel et al., 1998; De Bernardis et al., 2018). Alterations in the vaginal microbiome by changes in one of the factors listed above may result in the loss of beneficial bacteria such as *Lactobacilli*, which may lead to dysbiosis and subsequent candidiasis. In a healthy female, *Lactobacillus* inhibits candida*l* growth by competing for nutrients, preventing adhesion, and excreting fungicidal compounds (Basson, 2000; Kaewsrichan et al., 2006; Forster et al., 2016).

*Lactobacilli* have also been shown to inhibit hyphal formation, which decreases the virulence of *Candida* (Noverr and Huffnagle, 2004; Nguyen et al., 2011). The excretion of butyric acid by the bacteria has been shown to inhibit germ-tube formation (Noverr and Huffnagle, 2004). This finding has been tested *in vivo* as well, where pretreatment of *Galleria mellonella* larvae resulted in improved survival after *C. albicans* infection (de Oliveira et al., 2017; Ribeiro et al., 2017; Rossoni et al., 2017). *Lactobacilli* also have an anti-inflammatory effect which decreases production of cytokines such as IL-1β, IL-6, and IL-8 (de Oliveira Perrucini et al., 2019). Interestingly, studies have shown that when *Candida* is in a state of overgrowth, it keeps the abundance of *Lactobacillus* under control so that it maintains its dominance (de Oliveira et al., 2017).

With increased understanding of the interplay between bacteria and fungi, *Lactobacilli* probiotic supplements are widely utilized in women who suffer from recurrent vaginal candidiasis (Tachedjian et al., 2017; Zhang Z. et al., 2018). The supplements can be administered orally or topically, and have been shown to increase the efficacy of antifungal treatments along with preventing relapse (Kovachev and Vatcheva-Dobrevska, 2015; Pendharkar et al., 2015; Tachedjian et al., 2017).

Not all bacteria interact antagonistically with *C. albicans*. *Streptococci* (specifically Group B) and *E. coli* interact synergistically with *Candida* and can lead to a variety of pregnancy related complications such as preterm birth, low birth weight, and sepsis (Cools et al., 2016). For this reason, pregnant women in the US are routinely screened for Group *B Streptococci* (GBS) and treated appropriately prior to delivery. Multiple studies have demonstrated the potentiating effect of GBS on *Candida* which leads to increased virulence of this pathogenic yeast by increasing the adhesion of *Candida* to the host cells, along with increasing inflammation (Cools et al., 2016; Pidwill et al., 2018; Yu et al., 2018).

#### Genitourinary Tract

Till recently, urine in healthy individuals without an infection was considered to be sterile (Proctor, 2011; Magistro and Stief, 2019). However, this idea was based primarily on culture based studies, which mainly evaluated aerobic, fast-growing uropathogens (Proctor, 2011). Due to this early perception, the evaluation of urine was not included in the Human Microbiome Project (Turnbaugh et al., 2007). However, recent advancement in microbiome analyses fueled by high-throughput DNA based techniques have not only proven that microbes exist in healthy human urine, but have also shown that multiple urologic conditions may be associated with dysbiotic microbial states (Siddiqui et al., 2011; Khasriya et al., 2013; Hilt et al., 2014; Pearce et al., 2014; Thomas-White et al., 2016).

Compared to some of the other niches described in this review, the urologic investigation of microbes lags behind. There is significant interpersonal variability in urinary microbiome based on various factors such as age, sex, hygiene, diet, and hydration status. Furthermore, the advancement in the domain has been hampered by the lack of standardized sampling methods along with variable protocol for genetic analysis (Aragon et al., 2018). In addition, the relatively low abundance of microbial species, specifically fungi, in urine poses a challenge in DNA extraction and amplification (Ackerman and Underhill, 2017). The majority of investigations primarily focus on the bacterial alterations in the urinary microbiome that has been correlated with conditions such as overactive bladder, urinary incontinence, interstitial cystitis, neuropathic bladder, sexually transmitted infections, and chronic prostatitis/chronic pelvic pain syndrome (Ackerman and Underhill, 2017). Dysbiotic states have also been demonstrated in urologic malignancies such as bladder and prostate cancer (Bucevic Popovic et al., 2018).

The only two fungi that are consistently identified in urine belong to the Saccharomycetes class, which are *Candida* and *Saccharomyces* (Pearce et al., 2014; Nickel et al., 2016). Some of the other fungal species that have been identified to be pathogenic to humans include *Cryptococcus, Aspergillus, Coccidioides, Histoplasma*, and *Blastomyces* (Enoch et al., 2006; Sanglard, 2016).

The dearth of literature related to mycobiome in the urologic realm underscores the importance of continued efforts in understanding the complex interplay between host, bacteria, and fungi. While patients and practitioners are utilizing probiotics to reduce risk of infection in patients with recurrent urinary tract infections, the literature lags behind the practice. With improved understanding, we may be able to identify ideal candidates who may benefit from probiotic treatments or those who may need microbiome guided therapeutic intervention that may restore a symbiotic state to prevent future recurrences.

## Immune System

It is essential that the host immune system not only defends against invasive fungal growth, but also permits colonization of fungal commensals (Iliev and Underhill, 2013). The host’s response to endogenous and exogenous insults is directly determined by the microbiome. Host cells contain pattern receptors that allow them to detect the pathogen-associated molecular patterns, which are fungal cell wall components. In doing so, immune response is activated, turning on downstream proinflammatory and antimicrobial signaling pathways (Westerberg and Voyack, 2013; Tipton et al., 2017). In microbiome dysbiosis, this mechanism is altered and leads to inhibition of physiological immune responses and progression of disease. As such, the crosstalk between the microbiome and the host’s immune system is vital for the continued maintenance of homeostasis (Oever and Netea, 2014).

There are several mechanisms by which the microbiome mediates host immune defense. One important mechanism is via competitive exclusion by residing bacteria in mucosal niches (Oever and Netea, 2014). By doing so, the microbiota is protecting the body against invasive microorganisms, such as fungal pathogens. This response is particularly crucial against the overgrowth of *Candida* in invasive candidiasis as well as in gastrointestinal candidiasis since the severity of this disease is dependent on the fungal abundance and load colonizing the mucosa (Oever and Netea, 2014). Modulation of host antifungal immune response via bacterial commensals has been demonstrated with *Lactobacillus*. *Lactobacillus* is positively correlated with protection of the vaginal mucosa and the maintenance of a healthy microenvironment (Bradford and Ravel, 2017). Studies have shown that the lack of *Lactobacillus* in the vagina is strongly correlated with urogenital infections and HIV (Drell et al., 2013; Brown et al., 2015; Bradford and Ravel, 2017). In such situations, use of probiotics to increase concentrations of *Lactobacillus* are beginning to show promise (Oever and Netea, 2014; Bradford and Ravel, 2017).

A pathologic condition where the lack of immune system can be most readily studied is HIV. Mycobiome status is severely shifted in immunocompromised patients, specifically HIV patients. In fact, many complications exhibited in HIV patients are a product of mycobiome disruptions. For example, oral candidiasis is the most common oral complication in HIV patients (Mukherjee et al., 2014; Brown et al., 2015; Chandra et al., 2015). A study conducted by Brown et al. (2015) analyzed and compared the fungal and bacterial composition of HIV subjects against controls. They found that *Pichia* was absent from all HIV samples but present in control samples. *Pichia* is known to inhibit *Candida* and the formation of biofilms via the release of secretory factors and nutrient competition (Mukherjee et al., 2014; Brown et al., 2015; Chandra et al., 2015). The authors therefore proposed that the lack of *Pichia* in the HIV cohort probably contributes to the fungal dysfunction that allows for oral candidiasis to develop (Mukherjee et al., 2014; Brown et al., 2015). Mukherjee et al. (2014) conducted *in vivo* testing in which mice treated with *Pichia* spent medium (PSM) exhibited significantly decreased infection score and fungal burden compared to untreated mice. This study identified one such treatment that has the potential to develop into a novel antifungal in treating oral candidiasis in HIV patients.

Despite availability of antiretroviral therapy, pulmonary diseases pose as significant comorbidities in HIV patients (Cui et al., 2015). Specifically, COPD has been linked to HIV though the cause is poorly understood (Cui et al., 2015). It is possible that increased susceptibility to fungal infection in HIV manifests as pulmonary disease in these patients. The most common fungus in HIV patients, with or without COPD, is *Pneumocystis* and it is the leading cause of pneumonia in HIV-infected and immunocompromised patients (Cui et al., 2015). Cui et al. (2015) found that *P*. *jirovecii* and *Candida lacerata* are two overrepresented fungal species in the lungs of HIV patients; both of these species are known lung pathogens.

The immunocompromised status of HIV patients predisposes them to complications and comorbidities like oral candidiasis, COPD, and pneumonia. The mycobiome is inherently involved in this process and manipulating the fungal abundance in such patients to restore mycological balance could potentially alleviate the side effects exhibited in HIV.

## Allergy

With the advances in next-gen sequencing, investigators have been able to evaluate the fungal bioburden found in patients who suffer from conditions that have been correlated to allergic reactions. One of these conditions is allergic rhinitis, which refers to the chronic inflammation of the nasal cavity. In their 2015 study, Jung et al. (2015) evaluated the mycobiome profile of individuals with allergic rhinitis compared to healthy controls. They found that both groups had a high level of *Malassezia* species. However, the abundance of specific *Malassezia* species (e.g., *M. globosa, M. restricta*) varied from person to person. Additionally, there was a trend toward greater fungal diversity in allergic rhinitis individuals compared to healthy controls albeit this was not statistically significant (Jung et al., 2015). These findings may be significant in offering therapeutic interventions to this commonly encountered condition, which leads to significant compromise in quality of life.

## Conclusion

It is clear that the mycobiome plays a critical role in health and disease. However, the crosstalk between fungi and bacteria may be just as important as the fungi present. The current limited interest in the crosstalk between fungi and bacteria is a direct consequence of an incomplete understanding of the mycobiome, and the complexities associated with studying these polymicrobial interactions. It is therefore imperative to fully analyze and establish the fungal niches within the body to gain a broader understanding of the functionality of the microbiome, as a whole. This is evident in multiple body sites.

More research is needed to fully understand the interplay between fungus, bacteria, and the host, but it is evident that fungus can have a profound impact on health and disease. With the advancement in our sequencing technologies, and further elucidation of the organisms present in various niches, it is important to place a concerted effort in identifying approaches that may yield clinical efficacy in reestablishing a microbial diversity that prevents diseases and restores a healthy balance. These may include probiotics or dietary products based on our developing understanding of the inter-kingdom relationships between the microbiome and the mycobiome.

## Author Contributions

All authors involved equally in the conceiving of this project, writing, and editing.

## Conflict of Interest

The authors declare that the research was conducted in the absence of any commercial or financial relationships that could be construed as a potential conflict of interest.

## Publisher’s Note

All claims expressed in this article are solely those of the authors and do not necessarily represent those of their affiliated organizations, or those of the publisher, the editors and the reviewers. Any product that may be evaluated in this article, or claim that may be made by its manufacturer, is not guaranteed or endorsed by the publisher.

## Abbreviations

ITS: Internal transcribed spacer
OUT: Operational taxonomic units
CD: Crohn’s disease
CF: Cystic fibrosis
EPS: extracellular polysaccharide
GBS: *Group B Streptococci*.
